# TKI sensitivity patterns of novel kinase-domain mutations suggest therapeutic opportunities for patients with resistant ALK+ tumors

**DOI:** 10.18632/oncotarget.8173

**Published:** 2016-03-18

**Authors:** Amit Dipak Amin, Lingxiao Li, Soumya S. Rajan, Vijay Gokhale, Matthew J. Groysman, Praechompoo Pongtornpipat, Edgar O. Tapia, Mengdie Wang, Jonathan H. Schatz

**Affiliations:** ^1^ Department of Medicine, Division of Hematology-Oncology, Sylvester Comprehensive Cancer Center, University of Miami Miller School of Medicine, Miami, FL, USA; ^2^ Sheila and David Fuente Graduate Program in Cancer Biology, University of Miami Miller School of Medicine, Miami, FL, USA; ^3^ BIO5 Institute, University of Arizona, Tucson, AZ, USA; ^4^ Department of Pharmacology and Toxicology, University of Arizona, Tucson, AZ, USA; ^5^ Undergraduate Biology Research Program, University of Arizona, Tucson, AZ, USA; ^6^ Cancer Biology Graduate Interdisciplinary Program, University of Arizona, Tucson, AZ, USA

**Keywords:** anaplastic lymphoma kinase, drug resistance, crizotinib, ceritinib, alectinib

## Abstract

The anaplastic lymphoma kinase (ALK) protein drives tumorigenesis in subsets of several tumors through chromosomal rearrangements that express and activate its C-terminal kinase domain. In addition, germline predisposition alleles and acquired mutations are found in the full-length protein in the pediatric tumor neuroblastoma. ALK-specific tyrosine kinase inhibitors (TKIs) have become important new drugs for ALK-driven lung cancer, but acquired resistance via multiple mechanisms including kinase-domain mutations eventually develops, limiting median progression-free survival to less than a year. Here we assess the impact of several kinase-domain mutations that arose during TKI resistance selections of ALK+ anaplastic large-cell lymphoma (ALCL) cell lines. These include novel variants with respect to ALK-fusion cancers, R1192P and T1151M, and with respect to ALCL, F1174L and I1171S. We assess the effects of these mutations on the activity of six clinical inhibitors in independent systems engineered to depend on either the ALCL fusion kinase NPM-ALK or the lung-cancer fusion kinase EML4-ALK. Our results inform treatment strategies with a likelihood of bypassing mutations when detected in resistant patient samples and highlight differences between the effects of particular mutations on the two ALK fusions.

## INTRODUCTION

Anaplastic lymphoma kinase (ALK) [1] is an important therapeutic target in cancer, despite the function of the wild-type protein being poorly understood. While having key roles in early nervous system development [2], the levels of wild-type ALK subsequently drop off and remain at low levels for the remainder of life. Compounding ALK's enigmatic nature, the mammalian ligand(s) responsible for its activation are still up for debate [3–6]. Germline and acquired mutations in wild-type ALK, however, are found in the childhood cancer neuroblastoma [7–10]. Furthermore, chromosomal rearrangements fusing ALK's C-terminal kinase domain to a number of constitutively expressed genes result in malignant transformation through activation of multiple oncogenic signaling pathways [11, 12].

In *NPM-ALK*, identified in 1994 [13], *ALK* is fused to the constitutively expressed nucleophosmin gene [14]. Approximately 70% of anaplastic large cell lymphomas (ALCL) are positive for this or similar fusions [15]. In fact, it was through the discovery of this fusion that *ALK* was originally cloned [13]. Subsequently, in 2007, *ALK* was found fused to echinoderm microtubule associated protein-like 4 (*EML4*) yielding the fusion kinase EML4-ALK seen in approximately 3–5% of non-small cell lung cancers (NSCLC) [16, 17]. Several other ALK fusions since have been identified, including the *RANBP2* (RNA binding protein 2)-*ALK* fusion seen in inflammatory myofibroblastic tumor (IMT, reviewed in [14]).

Since the highly successful use of imatinib and other BCR-ABL tyrosine kinase inhibitors (TKIs) against chronic myeloid leukemia [18], there have been great efforts to find inhibitors that turn off other such kinases [19]. In 2011, a mere four years after the discovery of EML4-ALK, the U.S. FDA approved the dual ALK/MET TKI crizotinib for ALK+ NSCLC. While initial response to crizotinib may be strong [20–24], patients inevitably succumb due to acquired resistance through multiple mechanisms, including kinase-domain mutations, prompting development of newer generation inhibitors (Table 1).

**Table 1:** Kinase domain mutations leading to acquired resistance

Cell line	Observed mutation from this study	Variants observed by others	Disease observed in	Reported resistance phenotype	Reference
SUP-CR500-2	I1171S	I1171S	NPM-ALK ALCL	ASP-3026-R^*^	[39]
			EML4-ALK NSCLC	Crizotinib-R Alectinib-R	[38]
		I1171T	NPM-ALK ALCL	Crizotinib-R Ceritinib-R Alectinib-R AP26113-R ASP3026-R	[39, 46]
			EML4-ALK NSCLC	Crizotinib-R Ceritinib-S Alectinib-R	[36, 43–45, 47, 48]
		I1171N	NPM-ALK ALCL	Crizotinib-R Ceritinib-R Alectinib-R AP26113-R ASP3026-S	[39, 41, 42]
			EML4-ALK NSCLC	Alectinib-R	[38, 45]
			Neuroblastoma	Somatic	[10, 37, 70]
		I1171H	EML4-ALK NSCLC	Crizotinib-R	[36]
SUP-LR150	F1174L	F1174L	EML4-ALK NSCLC	Crizotinib-R Alectinib-S	[55, 57, 71]
			RANBP2-ALK IMT	Crizotinib-R	[56]
			Neuroblastoma	Somatic	[7–10, 37, 54, 72]
		F1174V	NPM-ALK ALCL	Crizotinib-S Ceritinib-S Alectinib-R AP26113-S ASP3026-R	[60]
			EML4-ALK NSCLC	Crizotinib-R Ceritinib-R	[43, 44, 47, 58]
			Neuroblastoma	Somatic	[7, 9, 10, 37]
		F1174C	NPM-ALK ALCL	Alectinib-R	[46]
			EML4-ALK NSCLC	Crizotinib-R Ceritinib-R	[36, 47]
			Neuroblastoma	Somatic	[7, 9, 10, 37]
		F1174I	NPM-ALK ALCL	Crizotinib-S Ceritnib-S Alectinib-S AP26113-S ASP3026-R	[39]
			Neuroblastoma	Somatic	[10, 37, 62]
		F1174S	Neuroblastoma	Somatic	[73]
DHL1-CR500	R1192P	R1192P	Neuroblastoma	Germline	[10]
		R1192Q	Uterine leiomyosarcoma		[74]
DHL1-LR150	T1151M	T1151M	Neuroblastoma	Germline	[8, 62]
		T1151R	Neuroblastoma	Germline	[75]
		T1151K	EML4-ALK NSCLC	Crizotinib-R	[36]
		1151Tins	EML4-ALK NSCLC	Crizotinib-R Ceritinib-R AP26113-R ASP3026-R	[25, 47, 58, 61]
DHL1-CR500-2	G1269A	G1269A	NPM-ALK ALCL	Crizotinib-R Ceritinib-S Alectinib-R AP26113-S ASP3026-R	[64]
			EML4-ALK NSCLC	Crizotinib-R Ceritinib-S Alectinib-R [64] Alectinib-S [48, 76] AP26113-S ASP3026-R	[47, 48, 58, 63, 64, 66, 76]
		G1269S	EML4-ALK NSCLC	Crizotinib-R	[36, 55]

Abbreviations: ALCL = Anaplastic Large Cell Lymphoma; NSCLC = Non-Small Cell Lung Cancer; IMT = Inflammatory Myofibroblastic Tumor; R = Resistant; S = Sensitive.

* Seen by ultra-deep sequencing at low frequency.

Here we assess the resistance/sensitivity profiles of mutations that arose in patient derived-cell ALCL lines continually exposed to either crizotinib or the 2nd generation ALK/IGF-1R inhibitor ceritinib (LDK378; FDA approved in 2014 for treating ALK+ NSCLC patients who failed crizotinib) [25]. Each mutation was profiled against six ALK TKIs – crizotinib, ceritinib, alectinib (which recently received FDA approval for treating ALK+ NSCLC patients who failed crizotinib [26]), AP26113 (brigatinib; a dual ALK/EGFR inhibitor in phase I/II trials that received FDA breakthrough therapy designation in 2014 [19]), ASP3026 (an ALK TKI in phase I trials [27, 28]) and AZD3463 (a dual ALK/EGFR inhibitor in preclinical development) [19, 29, 30].

## RESULTS

**Figure 1:**
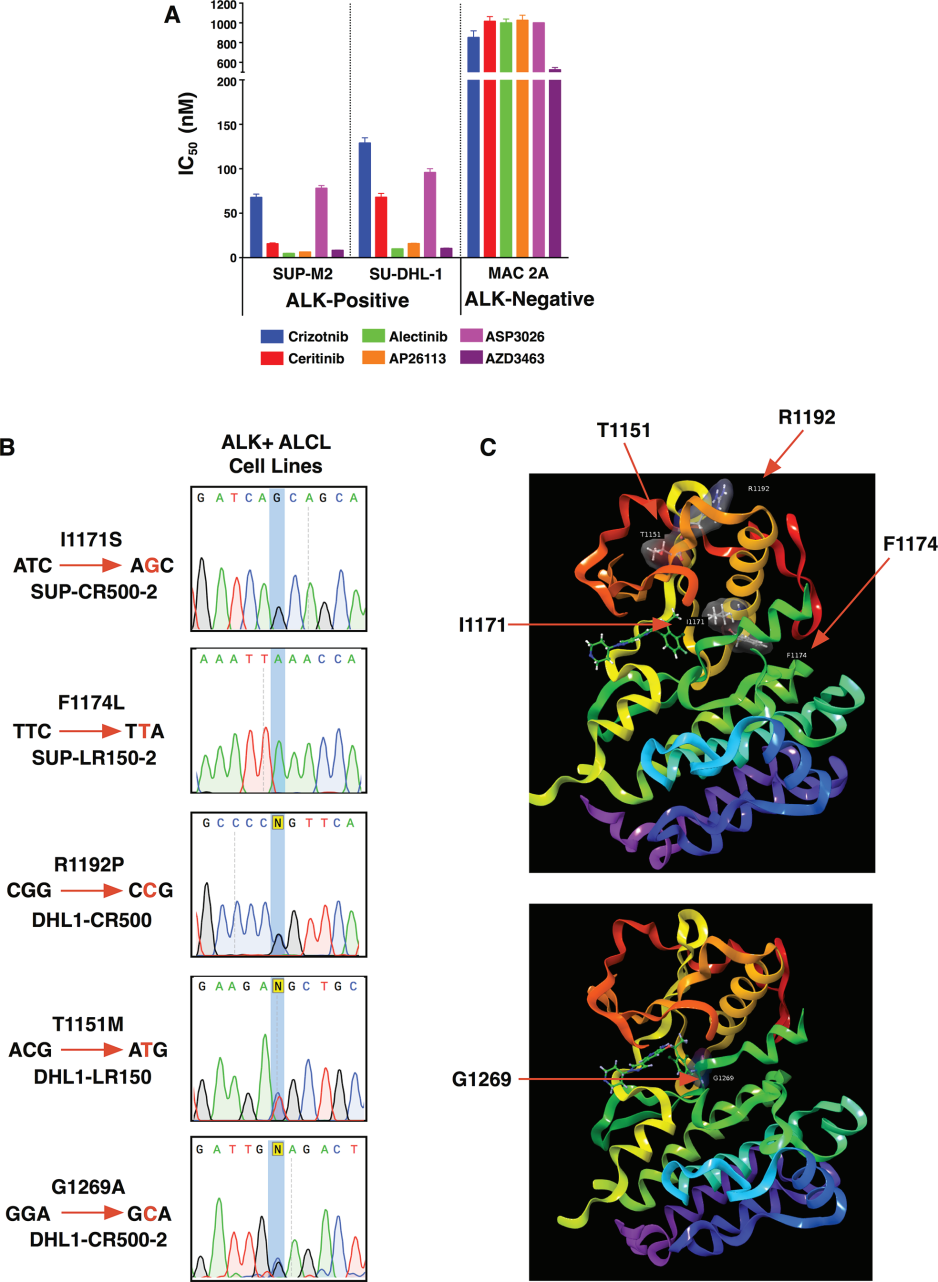
Acquired resistance mutations in patient-derived ALK+ ALCL cell lines (**A**) IC50s of parental ALK+ ALCL cell lines (SUP-M2 and SU-DHL-1) as well as an ALK-negative ALCL line (MAC-2A). Mean ± SEM for quadruplicates. (**B**) Sanger sequencing identifying each resistance mutation in cell lines. (**C**) Location of the five mutations identified in this study with respect to the ALK kinase domain shown as ball and stick models with associated surfaces colored by atoms.

The ALK+ ALCL cell lines SUP-M2 and SU-DHL-1, which are highly sensitive to ALK inhibition (Figure 1A), were selected in increasing concentrations of either crizotinib or ceritinib to investigate mechanisms of acquired resistance. We previously showed resistance initially was caused by increased *NPM-ALK* expression in all of these selections, and that this ALK up-regulation induced TKI-dependency as drug withdrawal resulted in the death of resistant cells [31]. Individual subclones, however, were able to grow again without ALK inhibitor following prolonged passaging, leading to normalization of *NPM-ALK* expression. These lines were named after their respective parent lines (SUP or DHL1), the inhibitor they were grown in (CR for crizotinib resistance, LR for ceritinib (LDK378) resistance), and the top nanomolar concentration in which they were able to proliferate during selection. Despite restoration of baseline *NPM-ALK* expression each line still exhibited varying degrees of persistent TKI resistance. Sequencing of the ALK TKD by Sanger and deep sequencing methods had suggested second-site mutations could be driving resistance, but we did not further characterize these initial findings. For this report, we maintained resistant clones in their top TKI concentration and then twice repeated Sanger sequencing of cDNA amplified from *NPM-ALK* mRNA. This detected a single second-site mutation in each resistant sub-clone (Figure 1B). Two of the mutations (I1171S from SUP-CR500-2 and F1174L from SUP-LR-2) were present as single peaks in the sequencing, indicating homogeneous populations in the sub-clones following drug selections. The other three mutations (R1192P from DHL1-CR500, T1151M from DHL-LR150, and G1269A from DHL1-CR500-2) appeared together with underlying wild-type peaks, indicating heterogeneous cell populations. While some of these mutations have been observed previously in the context of ALK-fusion cancers, we characterize two novel mutations that thus far have only been observed in neuroblastoma – T1151M and R1192P – and two mutations not previously characterized in ALK+ ALCL (Table 1). Each mutation was modeled on an X-ray structure of the ALK kinase domain (Figure 1C; discussed further below).

**Figure 2:**
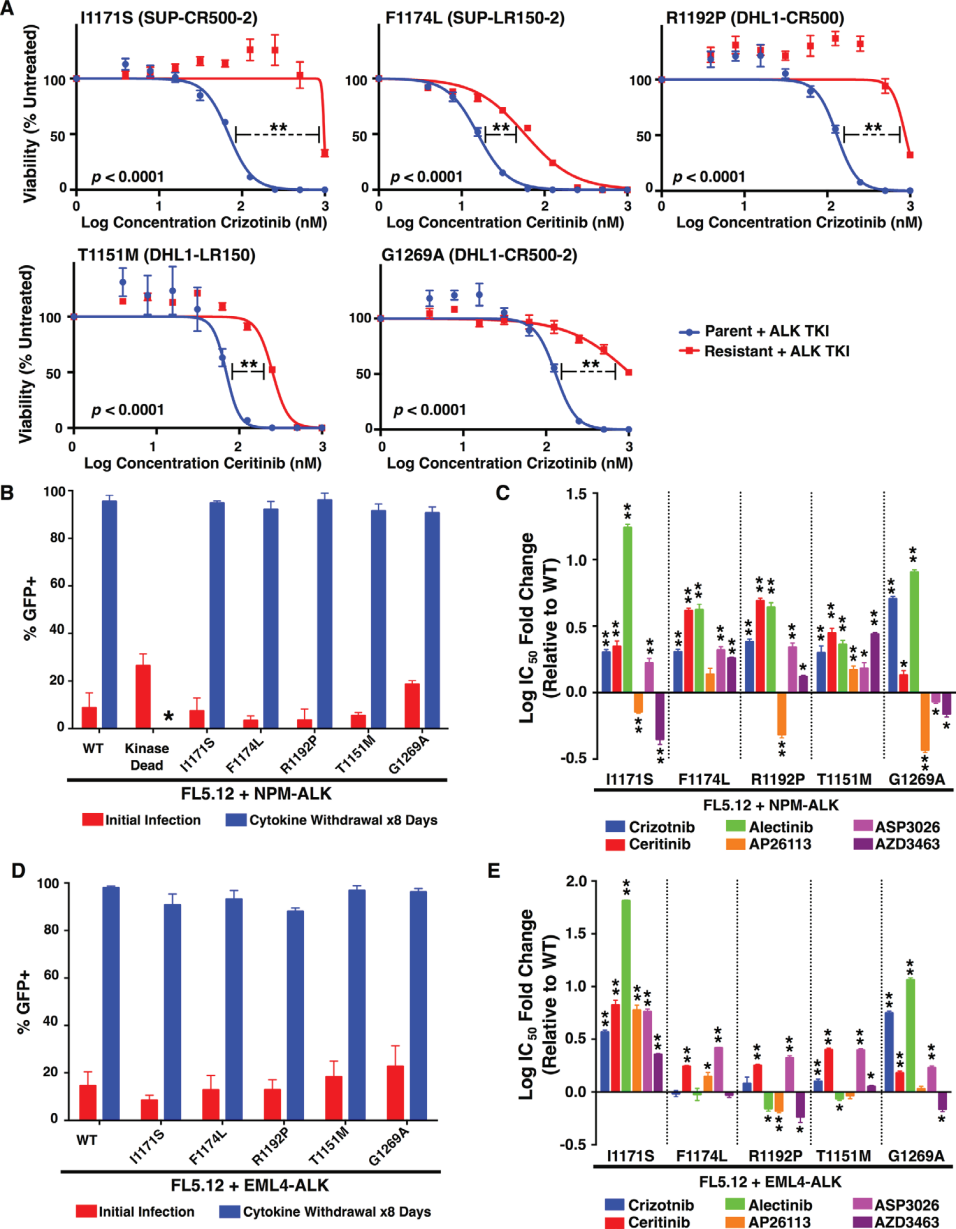
Resistance profiles of ALK mutations against six ALK TKIs (**A**) Cell viability assays (with denoted IC50s) for each resistant line compared to the parental line from which they were derived. (**B**) Cellular transformation of FL5.12 cells infected with an MSCV-based vector co-expressing GFP and wild-type or mutant NPM-ALK constructs upon cytokine withdrawal. The kinase-dead mutant was unable to survive in the absence of cytokine (*). (**C**) IC50's for each FL5.12 NPM-ALK construct against six ALK TKIs (see also Supplementary Figure S2). (**D**) Same as (B) but with mutations created in a retroviral vector containing EML4-ALK. (**E**) IC50's for each FL5.12 EML4-ALK construct against six ALK TKIs (see also Supplementary Figure S4). Mean ± SEM for quadruplicates (A, C and E) or triplicates (B and D). (A, C and E) Unpaired two-tailed *t*-test was performed using GraphPad Prism version 6 to compare the IC50s for each mutant to their respective parental (A) or wild-type (C, E) cells. *p < 0.05, **p < 0.001.

We first compared each subclone to its respective parent line for sensitivity to the TKI in which it had been selected (Figure 2A; Table 2). In all cases, subclones were significantly less sensitive, as determined by a highly significant increase in IC50, but additional factors could have arisen during selections to promote resistance. Furthermore, three of the five mutations were present in heterogeneous populations of cells also containing the wild-type NPM-ALK (as discussed above; Figure 1B). Therefore, to isolate the specific effect of each identified ALK-kinase mutation, we employed IL3-dependent FL5.12 murine pro-B cells as an independent system [32]. We generated each mutation through site-directed mutagenesis in *NPM-ALK* cloned into a GFP co-expressing MSCV-based vector (Supplementary Figure S1). Retroviral introduction of wild-type NPM-ALK or mutants, followed by IL3 withdrawal, transformed the FL5.12 cells from cytokine-dependence to oncogene-dependence (Figure 2B). Transformed lines withdrawn from IL3 proliferate only as 100% GFP+ clones, and dependence on NPM-ALK is further demonstrated by the failure of kinase-dead NPM-ALK or empty vector (not shown) to permit cytokine-independent growth. We then assessed sensitivity of lines transformed by each mutant to six TKIs in comparison to those transformed by wild-type NPM-ALK (Figure 2C; Supplementary Figure S2; Table 3). All five mutations exhibited significant cross-resistance to the three approved inhibitors, crizotinib, ceritinib, and alectinib. The two mutations derived from lines that had been selected in the second-generation inhibitor ceritinib, T1151M and F1174L, were pan-resistant to all five TKIs, though degree of resistance varied (discussed below). The other three mutations, from crizotinib-selected lines, remained sensitive to at least one other inhibitor. This independent system demonstrates resistance in ALK+ ALCL lines is driven at least in part by acquired ALK kinase-domain mutations, and confirms previous observations that mutations arising in response to one drug may affect multiple inhibitors of the same target.

**Table 2:** IC50s of each resistant line compared to the parent line from which they were derived

		Cell Line													
		SUP-M2		SU-DHL-1		I1171S SUP-CR500-2		F1174L SUP-LR150-2		R1192P DHL1-CR500		T1151M DHL1-LR150		G1269A DHL1-CR500-2	
		IC50	Fold Change	IC50	Fold Change	IC50	Fold Change	IC50	Fold Change	IC50	Fold Change	IC50	Fold Change	IC50	Fold Change
TKI	Crizotinib	67.75	1	128.9	1	878.03	12.96	-	-	887.18	6.88	-	-	933.275	7.24
	Ceritinib	15.57	1	67.94	1	-	-	57.69	3.7	-	-	249.98	3.68	-	-

**Table 3:** FL5.12 NPM-ALK mutant IC50s

		FL5.12 NPM-ALK											
		WT		I1171S		F1174L		R1192P		T1151M		G1269A	
		IC50	Fold Change	IC50	Fold Change	IC50	Fold Change	IC50	Fold Change	IC50	Fold Change	IC50	Fold Change
TKI	Crizotinib	171.85	1	345.03	2.01	347.63	2.02	421.5	2.45	346.65	2.02	871.75	5.07
	Ceritnib	34.03	1	75.8	2.23	140.45	4.13	167.08	4.91	95.78	2.81	46.04	1.35
	Alectinib	11.13	1	192.5	17.29	46.43	4.17	48.84	4.39	25.42	2.28	89.27	8.02

To further identify the potential clinical implications of each mutation, and to assess whether the effects of each mutation are fusion-specific, we employed an additional *in vitro* model. Each mutation was generated via site-directed mutagenesis in *EML4-ALK*, the most common ALK fusion detected in NSCLC, and cloned into the same GFP-expressing MSCV-based vector backbone described above (Supplementary Figure S3). Once again, using FL5.12 murine pro-B cells, retroviral introduction of each *EML4-ALK* mutant construct and subsequent IL3 withdrawal resulted in cytokine-independent, oncogene-dependent cellular transformation (Figure 2D). The resistance/sensitivity profiles of each *EML4-ALK* mutant versus wild-type *EML4-ALK* to the same panel of TKIs were assessed in the same manner as above (Figure 2E; Supplementary Figure S4; Table 4). For mutations previously observed in ALK TKI resistance models, our results were largely consistent with individual findings reported by others (Table 1). We observed some important differences, however, between the effects of particular mutations on NPM-ALK vs. EML4-ALK (compare Figure 2E and Figure 2C). In particular, R1192P and T1151M – never before detected in resistant EML4-ALK-driven cell lines or patient samples – had substantially greater effect on NPM-ALK in promoting resistance than on EML4-ALK. These findings among others highlight potentially clinically important differences mediated by ALK's fusion partner affecting selection by tumors for particular mutations (see discussion).

**Table 4:** FL5.12 EML4-ALK mutant IC50s

		FL5.12 EML4-ALK											
		WT		I1171S		F1174L		R1192P		T1151M		G1269A	
		IC50	Fold Change	IC50	Fold Change	IC50	Fold Change	IC50	Fold Change	IC50	Fold Change	IC50	Fold Change
TKI	Crizotinib	70.59	1	260.38	3.7	68.14	0.97	86.27	1.25	89.07	1.27	395.2	5.62
	Ceritnib	9.6	1	64.69	6.79	16.86	1.76	16.96	1.79	24.24	2.53	14.57	1.52
	Alectinib	3.46	1	224.28	64.98	3.35	0.98	2.55	0.70	3.00	0.87	40.09	11.6
	AP26113	4.13	1	24.87	6.09	5.79	1.42	2.7	0.66	3.81	0.93	4.4	1.08
	ASP3026	42.21	1	245.13	5.83	110.97	2.63	90.59	2.11	105.73	2.51	71.79	1.71
	AZD3463	16.79	1	38.27	2.28	15.83	0.94	10.16	0.59	18.99	1.13	11.62	0.69

**Figure 3:**
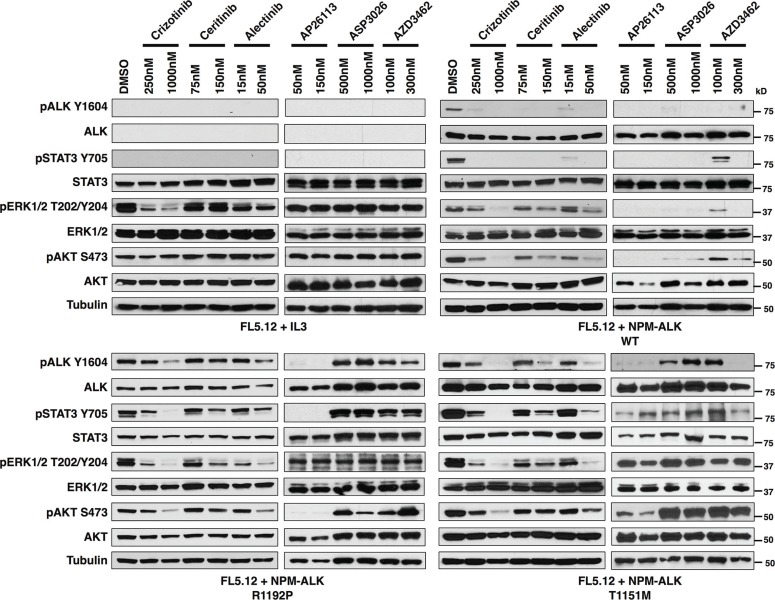
Activated ALK and downstream signaling is preserved in the novel ALK-fusion mutations, R1192P and T1151M Immunoblotting for ALK and downstream signaling targets at the indicated concentrations of six ALK TKIs for FL5.12 cells (A), FL5.12 cells infected with an MSCV-based vector co-expressing GFP and wild-type NPM-ALK (B), NPM-ALK R1192P (C) or NPM-ALK T1151M (D).

We next assessed through immunoblotting the signaling consequences in FL5.12 cells of NPM-ALK-R1192P and T1151M, the two mutations not previously described as fusion ALK-kinase resistance mutations (Figure 3). Results suggest a particularly strong resistance phenotype of R1192P to all the inhibitors except AP26113, in line with the IC50 data. The effect of T1151M, meanwhile, was weaker, with higher concentrations of all drugs except ASP3026 overcoming its effects on ALK phosphorylation. We note that pERK levels decreased in crizotinib-treated FL5.12 cells not transfected with NPM-ALK (growing in IL3), and pERK is correspondingly more crizotinib sensitive in all the transformed lines as well. This suggests an off-target effect such as this drug's known activity against MET [33]. Similar to our published findings, phosphorylation of ERK and AKT were overall variable in these results, providing inconsistent markers of drug potency [31]. STAT3 activation, however, (indicated by phosphorylation at Y705), which is known to be ALCL's core survival pathway was strongly consistent with drug potency and ALK phosphorylation status [34, 35]. We therefore confirm drug-resistant ALK activation promoting ALCL's core survival pathway mediated by NPM-ALK-R1192P and T1151M, consistent with viability data.

## DISCUSSION

Resistance to ALK-kinase inhibition in lung cancer is described in several published studies that employed cell lines, patient samples, or both. Our goal has been to understand resistance in ALK+ ALCL, which is less well studied. We previously reported resistance driven by *NPM-ALK* up-regulation, which also paradoxically drives dependence on continued inhibitor exposure, defining for the first time oncogene overdose by ALK over-activation [31]. Here, we examine five *NPM-ALK* kinase domain mutations that drive resistance in ALCL in an absence of over-expression. Two of these, R1192P and T1151M, are previously unreported as ALK TKI resistance mutations. We evaluated mutations in an independent system permitting isolation of mutation effect from other factors that may arise in ALK-dependent cells continually exposed to inhibitors. These experiments showed R1192P, T1151M, and the other mutations in *NPM-ALK* adversely affected the activity of multiple ALK TKIs but also pointed to inhibitors for which sensitivity is maintained or even enhanced. Interestingly, cross-validation using the lung cancer-derived *EML4-ALK* fusion showed both similarities and key differences in the effect of mutations on particular drugs. Our study therefore characterizes novel *NPM-ALK* TKI resistance mutations, providing guidance for appropriate choice of alternate therapies as more of these drugs move toward regulatory approval. It also highlights key differences in the effect of mutations on the ALK kinase domain's sensitivity to inhibition depending on its fusion partner. For example, the fact that R1192P and T1151M have not previously been detected in resistant EML4-ALK-driven systems is not surprising since their effect on drug activity was significantly less for that kinase than for NPM-ALK. The profile of ALK TKI resistance mutations likely to arise in a tumor, therefore, is inherently shaped by ALK's fusion partner, as should choices of alternative therapy.

### I1171S

I1171S previously was seen in an *in vitro* accelerated mutagenesis screen in ALK+ NSCLC using increasing concentrations of crizotinib [36] and somatic mutations in this residue also have been reported in neuroblastoma [10, 37]. Subsequently, this mutation was identified in an ALK+ NSCLC patient as conferring alectinib resistance [38]. Consistent with its original identification in EML4-ALK-driven systems, we find I1171S is potently resistance-conferring to FL5.12 cells dependent on this fusion. All six TKIs tested had significantly increased IC50s when tested vs. EML4-ALK-I117S compared to wild-type (Figure 2E). In NPM-ALK, I1171S promoted resistance to crizotinib, the drug used in selecting the line in which it was isolated, ceritinib, and again pronounced resistance to alectinib. Ultra-deep sequencing previously identified I1171S at very low frequencies as a candidate ASP3026-resistant mutation in ALK+ ALCL [39], which is also confirmed in our functional system. Interestingly, however, NPM-ALK-I1171S promoted no resistance, indeed increased sensitivity compared to wild type, to both AP26113 and AZD3463. The effect of a resistance mutation conferring sensitivity to other inhibitors is similar to a recent high-profile case report in which the L1198F mutation, providing resistance to the third-generation inhibitor lorlatinib, resulted in resensitization to crizotinib [40]. The novel finding in our study is that this effect was seen for NPM-ALK but not EML4-ALK. Our data do not identify the reason for differential the effects of I1171S on AP26113 and AZD3463 (structurally similar compounds that also inhibit EGFR) between NPM-ALK and EML4-ALK. Given the FL5.12 system used is identical in all ways other than ALK's fusion partner, the effect is likely on-target, mediated by subtle ATP binding-pocket differences between the two fusion kinases.

Several other amino acid substitutions occur at I1171 residue, all promoting resistance to both crizotinib and alectinib regardless of whether their characterization was in the context of NSCLC or ALCL (Table 1) [39, 41–46]. There are some differences in TKI resistance between our findings for I1171S and other amino acid substitutions at this residue. For instance, in lymphoma studies, I1171N and I1171T are reported to be AP26113-resistant [39, 41], and while both appear resistant to ceritinib in NPM-ALK [39, 41], they show sensitivity in EML4-ALK [43, 44, 47, 48]. Further variability exists for both I1171N and I1171T, with the former being ASP3026-sensitive [39, 42] and the latter resistant [39] in NPM-ALK.

I1171 is part of the hydrophobic regulatory spine (R-spine), along with residues C1182, F1271 (DFG motif), H1247 (HRD motif) and D1311 (F-helix), which connects the two major ALK TKD lobes. Mutations in I1171 are believed to lock ALK in its active conformation, accelerating its activation through autophosphorylation [37, 43, 49–52]. Additionally, mutations at this residue decrease stability of inhibitor binding at the DFG motif, and, in the case of at least I1171N, modify the structure of the kinase-inhibitor complex [41]. Our data suggest NPM-ALK+ ALCL patients who develop resistance due to I1171S could benefit from treatment using either AP26113 or AZD3463, as both overcome this mutation in our independent system. Other inhibitors may be necessary in the context of EML4-ALK.

### F1174L

The phenylalanine residue at position 1174, located at the end of the αC helix amongst a highly hydrophobic cluster of residues, maintains autoinhibitory interactions when ALK is in its inactive conformation [37, 49, 51] and is a mutational hotspot within the ALK kinase domain [53]. We found F1174L in a cell line grown in increasing concentrations of ceritinib. It is a frequent somatic mutation in neuroblastoma and increases ALK's affinity for ATP, despite not directly contacting the ATP pocket. Accordingly, resistance is not due to steric hindrance of drug binding, but rather promotion of ALK's active conformation indicated by accelerated autophosphorylation [10, 37, 54]. F1174L therefore acts as both a TKI-resistance and an activating mutation [7–10, 37, 54]. In agreement with our observations for NPM-ALK (Table 1; Figure 2C; Supplementary Figure S2), F1174L results in resistance to crizotinib in both ALK+ NSCLC and ALK+ IMT [55–57], although we did not observe a significant change in resistance in our EML4-ALK *in vitro* assay (*p* = 0.4846; Table 4; Figure 2E; Supplementary Figure S4). In contrast to previous reports in ALK+ NSCLC [56–58] along with our EML4-ALK *in vitro* assay, we observe resistance to alectinib, which is more in agreement with this mutation favoring ATP binding over inhibitor binding, and suggests potential differences in the conformational changes induced by a leucine substitution at this residue based on ALK's fusion partner. Studies involving other amino acid substitutions at position 1174 support this. Whereas F1174C and F1174V are resistant to crizotinib and ceritinib in ALK+ NSCLC cell line models and patient samples [36, 47, 59], F1174V and F1174I are sensitive to both TKIs in ALK+ ALCL [39, 60].

Furthermore, despite F1174C and F1174V showing resistance to alectinib in ALK+ ALCL [46, 60], this drug is able to overcome an isoleucine substitution in the same model [39]. Additionally, while we observe a small fold-increase in the IC50 for AP26113 (1.4-fold) for this mutant compared to wild-type NPM-ALK, the increase is not statistically significant (*p* = 0.0513), and AP26113 has been reported to show sensitivity to both F1174V and F1174I [39, 60]. Our data for both fusions, in agreement with other studies in NPM-ALK, show ASP2036 is unable to overcome any amino acid substitutions at this location [39, 60]. Furthermore, while AP26113 may show slight efficacy in overcoming ALK+ ALCL resistance acquired by this mutation (*p* = 0.0513), it is predicted to be ineffective in ALK+ NSCLC, with the opposite being true for AZD3463 with respect to each disease. This further highlights the care required when considering treatment options to overcome resistance in patients, as both the specific substitution and the particular ALK-fusion may be key factors.

### R1192P

The R1192P mutation, residing in the N-lobe, is one of the most frequent germline mutations in neuroblastoma [10]. In a similar fashion to both I1171 and F1174 mutations, R1192P results in accelerated autophosphorylation of ALK's TKD [37]. In fact, this mutation is considered an exception, as most residues in the N-lobe have smaller impacts. We report here for the first time the identification of this mutation in any ALK-fusion cancer, in a cell line selected in crizotinib. Perhaps unsurprisingly, due to its ability to strongly turn on the kinase activity of ALK, all but one TKI proved to be ineffective at overcoming resistance to this mutation in NPM-ALK (Table 1; Figures 2C, 2D and 3; Supplementary Figure S2). The only exception, AP26113, may be an attractive therapeutic in the event of resistance by this mutation. The effect of this mutation was dramatically less vs. all drugs when it was introduced instead to EML4-ALK, likely explaining, as discussed above, why it has never been reported in resistant ALK+ lung cancer. Detailed structural determinations are outside the scope of this report, but it is interesting to note that all three mutations reported to activate ALK kinase rather than block drug binding to the ATP pocket – F1174L, R1192P, and T1151M (below) – have greater effect on drug activity when found in NPM-ALK than in EML4-ALK. Follow-up structural chemistry studies to this report may confirm and identify the basis for this effect.

### T1151M

T1151M was identified recently in neuroblastoma patients [37] and we present here the first report of this mutation in the ALK-fusion context, arising in a cell line grown in ceritinib. An amino acid insertion, 1151T*ins* is well-characterized as causing cross-resistance to several ALK TKIs in ALK+ NSCLC cell lines and patients [25, 47, 61], although one study reports that alectinib is effective against it [58]. Additionally, 1151T*ins* was reported in neuroblastoma [62]. Finally, the amino acid substitution T1151K was also found in an accelerated mutagenesis screen in ALK+ NSCLC assessing crizotinib resistance [36].

Mutations at residue 1151, located at the N-terminal lobe of the ALK catalytic domain, alter affinity of the mutated kinase for ATP, and diminish inhibitor binding through conformational changes, despite lying some distance from the ATP pocket [47, 61]. Furthermore, mutations in this domain lead to activation of the ALK TKD, albeit more modestly than I1171, F1174, or R1192 [37]. It is therefore perhaps unsurprising that 1151T*ins*, T1151K, or, in our case, T1151M in NPM-ALK, cause cross-resistance to several ALK TKIs due to the mutant kinase domain strongly favoring ATP binding, as confirmed by fold-changes in IC50 and western blotting (Table 1; Figure 2C and 2D; Supplementary Figure S2). Somewhat surprising is the lack of significant change in response to AP26113 (*p* = 0.2090) and sensitivity to alectinib (*p* = 0.0169; in agreement with Kodama et al. [58] as mentioned above) when compared to wild-type EML4-ALK, further highlighted the importance of the particular amino acid substitution, as well as the potential differences in protein folding dependent upon the fusion partner in question.

### G1269A

In agreement with our observations, this mutation resists crizotinib in both NPM-ALK (as expected as it was isolated from a cell line grown in crizotinib) and EML4-ALK, but is sensitive to AP26113 in both fusions [47, 63, 64]. A serine substitution at the same residue behaves similarly in EML4-ALK [36, 55]. G1269 lies in the ATP-binding pocket, making direct contact with crizotinib. Using computer modeling (Figure 1C), we observed that the dichlorofluorophenyl ring of crizotinib binds near G1269 (distance 3.49 Å between Ca G1269 and fluorine atom). G1269A causes a steric clash between the dichlorofluorophenyl ring and the alanine's methyl group, apparently sufficient to disrupt the drug's activities [47, 63, 65]. Much like T1151 mutations, G1269 mutations cause modest constitutive ALK tyrosine kinase activity [37].

While we observed resistance to ceritinib in both fusions (Table 1; Figure 2C; Supplementary Figure S2), others have shown that ceritinib effectively overcomes G1269A in both ALK+ NSCLC [25, 47] and ALK+ ALCL [64] due to it stabilizing ALK's conformational dynamics and exhibiting increased potency for this mutant over WT ALK [48]. Once again, the degree of resistance in our study was only mild (1.35-fold for NPM-ALK and 1.52-fold for EML4-ALK), albeit still statistically significant (*p* = 0.0018 and < 0.0001, respectively), which may explain this difference. Another discrepancy is with ASP3026, predicted to overcome resistance in NPM-ALK by our data but unable to do so in a separate study in both ALK+ NSCLC and ALK+ ALCL [64]. However, the sensitivity reported in our study is just barely significant (*p* = 0.0495), perhaps explaining this discrepancy. Additionally, while we and Fontana and colleagues [64] report that alectinib is unable to overcome G1269A in the context of either disease, two other studies showed a response to alectinib *in vitro*, in xenograft models, and in a patient harboring this mutation [58, 66]. Borderline results *in vitro* may therefore be less reliable predictors of responses *in vivo*. Indeed, others have shown some TKIs with IC50 shifts *in vitro* may still cause tumor regression *in vivo* [47]. Further trial and error, including studies in resistant patients, will be necessary to clarify the situation for some mutations. Our data suggest AZD3463 may be able to overcome G1269A in both fusion-cancers. Both ASP3026 and AZD3463 interact differently compared to crizotinib with the active site near G1269. The X-ray crystal structure of ASP3026 with the ALK kinase domain (PDB code: 2XB7) shows that the isopropylsulfone group interacts with lysine 1150 through hydrogen bonding. This bond draws ASP3026's binding away from G1269 (distance = 5.85 Å between G1269 Ca and methylene carbon), and it is expected that ASP3026 would bind G1269A mutant in similar fashion.

The ALK kinase has several mutational hotspots, such as F1174, F1245 and R1275 [53]. The identification of two mutations not previously reported in any malignancy driven by an ALK-fusion (T1151M and R1192P) is therefore highly informative. Despite a predilection for certain mutations conferring resistance, “novel” mutations at sites other than mutational hotspots can still occur, perhaps albeit at lower frequencies, as the tumor evolves in a desperate attempt to evade death. Our findings with mutations favoring ATP-binding and dampening inhibitor-binding and/or constitutively activating ALK's TKD, suggest newer competitive inhibitors are desperately needed to overcome acquired resistance. One such inhibitor, the first 3rd generation ALK (and ROS1) inhibitor PF-06463922 (lorlatinib), exhibits increased potency against F1174L, 1151Tins, I1171T and G1269A in preclinical EML4-ALK models [67, 68], with impressive anti-tumour activity observed in xenograft neuroblastoma models harboring F1174L [53]. Another new inhibitor, a structural analog of alectinib, JH-VIII-157-02, also shows great promise against a series of ALK resistance mutations, including G1269A, F1174L and 1151Tins [69]. Further development and screening of newer generation ALK inhibitors is highly important in overcoming resistance mutations in patients suffering from ALK-related malignancies.

## MATERIALS AND METHODS

### Cell lines, reagents and inhibitors

RPMI 1640 and penicillin/streptomycin (P/S) supplemented with 10% fetal bovine serum (FBS) for SU-DHL-1 and MAC 2A, or with 20% FBS for SUP-M2. Phoenix cells grown in DMEM plus 10% FBS and P/S. FL5.12 cells cultured in RPMI 1640 with 10% FBS, P/S, ± 10% WeHi-3B supernatant and murine IL3 (400 ρM, eBioscience). All lines purchased from DSMZ apart from FL5.12's (gift from Wendel lab). Crizotinib and alectinib were purchased from Selleck Chemicals; ceritinib (LDK378) was kindly provided by Novartis.

### Cell viability assays

3000 cells per well were seeded in serial dilutions of the indicated ALK TKIs. Viability was assessed after 72 hours (Cell Titer Glo^®^; Promega) by measuring luminescence on a BioTek Synergy HT plate reader. GraphPad Prism version 6 was used to calculate IC50s with non-linear curve-fit regression.

### Computer modeling

Images in Figure 1C were created using the X-ray structure of ALK kinase domain using the Maestro software (Schrodinger Inc.)

### Identification of kinase-domain mutations

RNA extraction (RNeasy^®^ Mini Ki; QIAgen) followed by cDNA synthesis (Taqman^®^ Reverse Transcriptase kit; Roche) was carried out two independent times for all resistant cell lines as well as their respective parental lines. The NPM-ALK fusion was PCR amplified (Primers - Forward: GTCCGCCTTCTCTCCTACCT, Reverse: TTGGCACAAAACAAAACGTG) on a BioRad T100 Thermal Cycler. The kinase domain was sequenced by Sanger sequencing. The sequences obtained for the resistant lines were aligned to their respective parental lines using the ClustalW online sequence alignment tool to identify base pair changes. The identified mutations were the same for both independent sets of sequencing.

### Protein extraction, quantification and immunoblotting

As described previously [31], loading 30 μg per lane, with all primary and secondary antibodies from Cell Signal Technology, developed using autoradiograph film (GeneMate).

### Site directed mutagenesis

Plasmid purification (PerfectPrep Spin Mini Kit; 5Prime) was carried out for MSCV-based -NPM-ALK and -EML4-ALK vectors mutated using site directed mutagenesis (QuikChange II XL; Agilent Technologies). Sanger sequencing was then used to confirm the presence of mutations in the ALK TKD.

### Transfections, infections and cellular transformation

As described previously [31] using an MSCV-based vector co-expressing GFP plus either wild-type NPM-ALK, wild-type EML4-ALK, each NPM-ALK mutation, the same mutations in EML4-ALK, or an NPM-ALK kinase dead mutation (K210R) as a negative control. Cellular transformation was assessed by flow cytometry (Guava EasyCyte).

## SUPPLEMENTARY MATERIALS FIGURES


