# Supplementary material for: TKI sensitivity patterns of novel kinase-domain mutations suggest therapeutic opportunities for patients with resistant ALK+ tumors
Source: Oncotarget. 2016 Mar 18;7(17):23715–29. doi: 10.18632/oncotarget.8173 (PMC5029658; doi:10.18632/oncotarget.8173)
Supplement: Supplementary file 1 [file oncotarget-07-23715-s001.pdf]

TKI sensitivity patterns of novel kinase-domain mutations suggest therapeutic opportunities for patients with resistant ALK+ tumors

Supplementary Materials

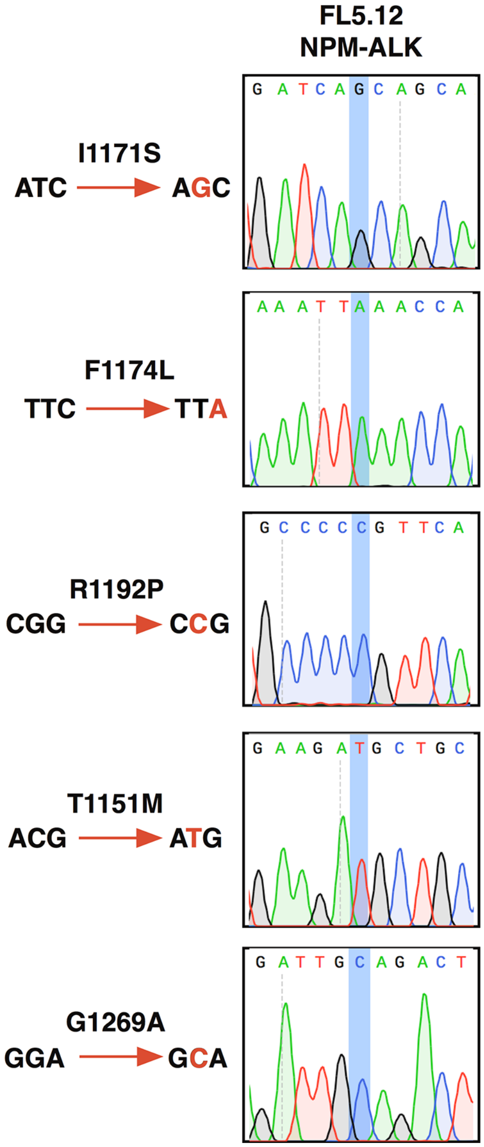

Supplementary Figure S1: Site directed mutagenesis to create each resistance mutation in a retroviral NPM-ALK vector. Site directed mutagenesis was followed by Sanger sequencing to confirm the presence of mutations in the ALK TKD.

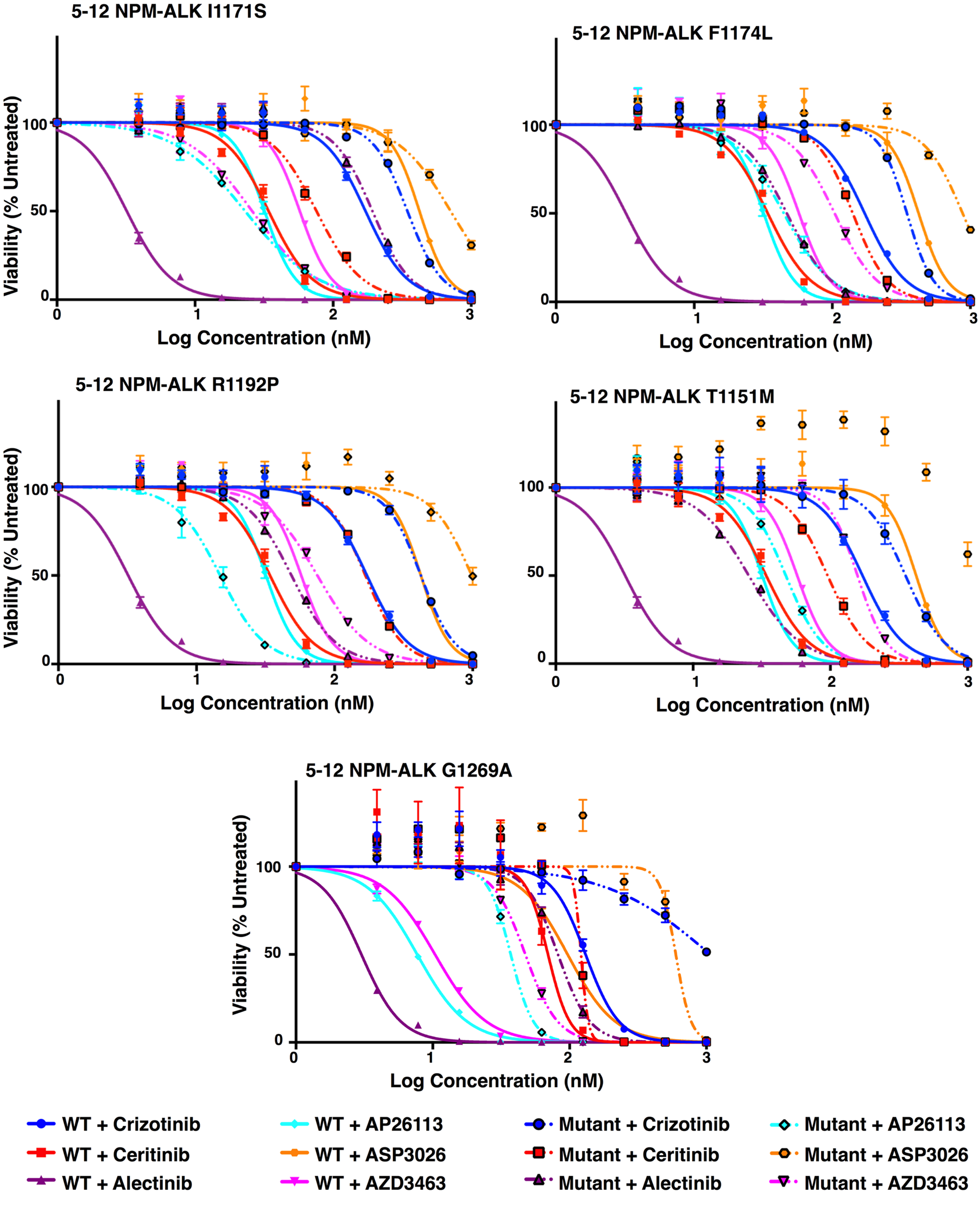

Supplementary Figure S2: Cell viability assays for each mutation isolated in an independent, transformed cell line against six ALK TKIs. Cell viability for each FL5.12 NPM-ALK mutated construct (See Figure 2C for IC50s). Mean ± SEM for quadruplicates.

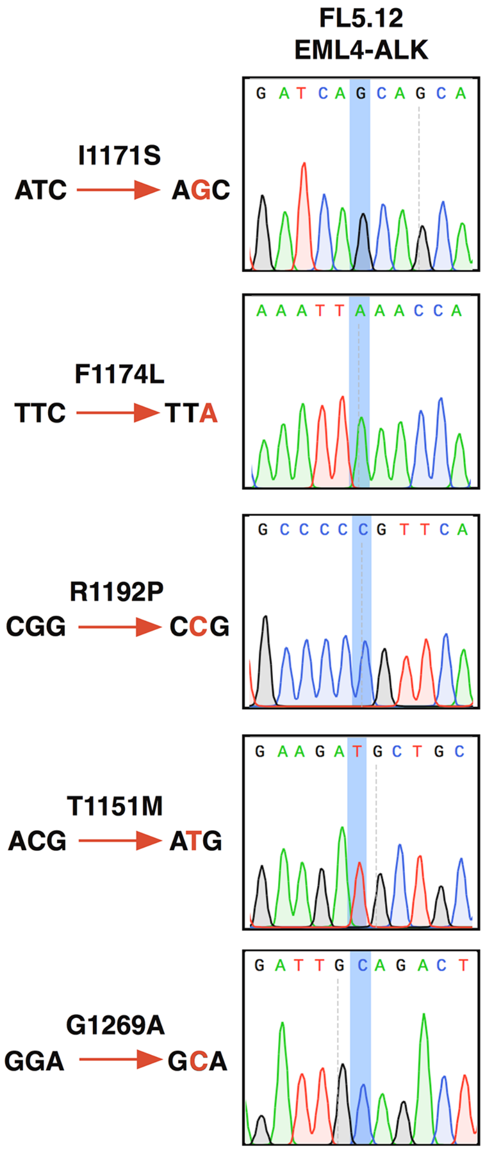

Supplementary Figure S3: Site directed mutagenesis to create each resistance mutation in a retroviral EML4-ALK vector. Site directed mutagenesis was followed by Sanger sequencing to confirm the presence of mutations in the ALK TKD.

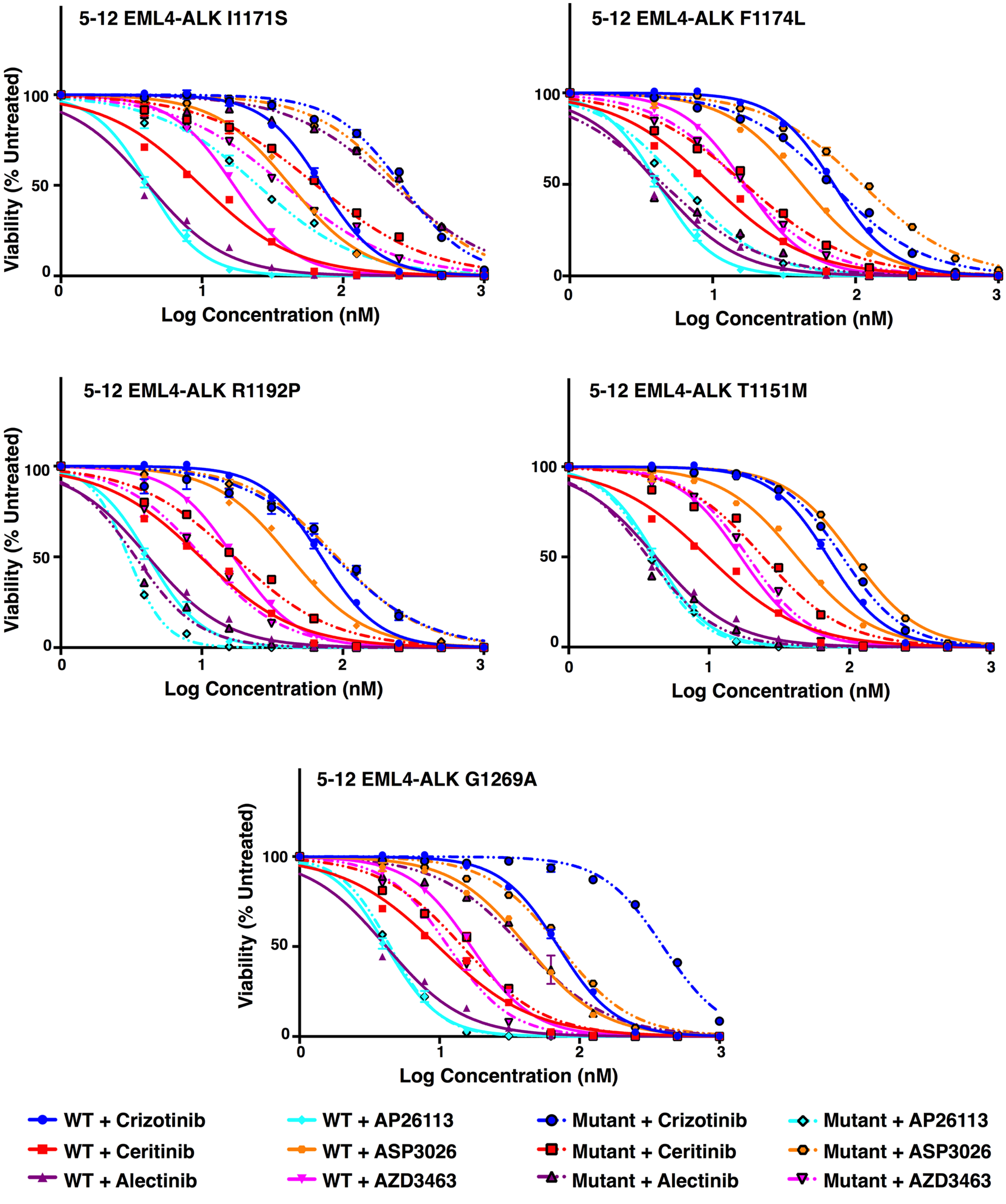

Supplementary Figure S4: Cell viability assays for each mutation isolated in an independent, transformed cell line against six ALK TKIs. Cell viability for each FL5.12 EML4-ALK mutated construct (See Figure 2E for IC50s). Mean ± SEM for quadruplicates.
